# Metallo-Graphene Nanocomposite Electrocatalytic Platform for the Determination of Toxic Metal Ions

**DOI:** 10.3390/s110403970

**Published:** 2011-04-01

**Authors:** Chandre M. Willemse, Khotso Tlhomelang, Nazeem Jahed, Priscilla G. Baker, Emmanuel I. Iwuoha

**Affiliations:** Sensor Lab, Department of Chemistry, University of the Western Cape, Bellville 7535, South Africa; E-Mails: 2607160@uwc.ac.za (C.M.W.); 3077260@uwc.ac.za (K.T.); pbaker@uwc.ac.za (P.G.B.); eiwuoha@uwc.ac.za (E.I.I.)

**Keywords:** Nafion-Graphene nanocomposite, mercury film, trace metals, square-wave anodic stripping voltammetry

## Abstract

A Nafion-Graphene (Nafion-G) nanocomposite solution in combination with an *in situ* plated mercury film electrode was used as a highly sensitive electrochemical platform for the determination of Zn^2+^, Cd^2+^, Pb^2+^ and Cu^2+^ in 0.1 M acetate buffer (pH 4.6) by square-wave anodic stripping voltammetry (SWASV). Various operational parameters such as deposition potential, deposition time and electrode rotation speed were optimized. The Nafion-G nanocomposite sensing platform exhibited improved sensitivity for metal ion detection, in addition to well defined, reproducible and sharp stripping signals. The linear calibration curves ranged from 1 μg L^−1^ to 7 μg L^−1^ for individual analysis. The detection limits (3σ blank/slope) obtained were 0.07 μg L^−1^ for Pb^2+^, Zn^2+^ and Cu^2+^ and 0.08 μg L^−1^ for Cd^2+^ at a deposition time of 120 s. For practical applications recovery studies was done by spiking test samples with known concentrations and comparing the results with inductively coupled plasma mass spectrometry (ICP-MS) analyses. This was followed by real sample analysis.

## 1. Introduction

Heavy metals such as cadmium and lead pose a detrimental risk to human health and the environment due to their toxicity; even exposure to minuscule quantities can be life threatening. For example, the toxicity of lead in humans mainly arises from its mimicking action of occupying the calcium binding sites on numerous calcium-dependent proteins in cells resulting in the corresponding impairment of physiological functions [1]. On the other hand other metals such as zinc are essential nutrients, but over or under exposure can also be toxic [2].

The search for a rapid, sensitive and simple analytical method for trace metal monitoring is needed. At present the more popular techniques for analyzing trace heavy metals are based on spectroscopic techniques, namely atomic absorption spectroscopy, inductively coupled plasma-atomic emission spectroscopy (ICP-AES) and ICP-mass spectrometry (ICP-MS). However, spectroscopic methods are expensive, their availability is limited, they are not well suited for *in situ* measurements and require complicated instrumentation. Electrochemical (EC) techniques on the other hand are one of the best methods for detecting metals owing to their low cost, high sensitivity and portability.

Amongst all of the EC techniques, electrochemical stripping analysis is recognized as a powerful tool for trace metal analysis [2]. This technique is capable of measuring four to six analytes in a sample simultaneously in the sub parts per billion (sub-ppb) range. The instrumentation is compact and has a low power demand (small carbon footprint) and, requires no special installation or additional instrumentation and is suitable for on-site and *in situ* analysis [3,4]. Although stripping analysis is not a panacea for trace metal analysis, it does offer an alternative method. Electrochemical stripping analysis can also be used in complex matrices such as the determination of lead and cadmium in human hair [5], determination of zinc in oyster tissue and sewage sludge [6], as well as for the determination of lead and copper in wine [7].

The use of chemically modified electrodes, heated electrodes, microwave electrodes and insonated electrodes in stripping analysis to improve the sensitivity of the sensing interface for metal ion analysis have also been investigated [8–11]. In addition, the development of nanotechnology offers greater potential of increased sensitivity in metals analysis especially when incorporating of carbon nanotubes [12,13], ordered mesoporous carbon [14], functionalized mesoporous silica electrode [15], nanocrystalline diamond thin-film electrode [16] and the thick film modified graphite containing electrode [17], has greatly improved stripping signals but new materials are still needed to develop highly sensitive and antifouling metal ion sensing platforms.

Due to its excellent electronic, thermal and mechanical properties graphene, a single atom thick sheet of hexagonally arrayed sp^2^ bonded carbon atoms, has recently been attracting a lot of attention since it was first produced experimentally in 2004. It is suggested to be a very important material in device applications. Li *et al.* developed a cadmium sensing platform comprising of a Nafion-G coating onto which a mercury film was deposited [18].

In this work the use of SWASV together with a Nafion-G nanocomposite in combination with an *in situ* generated mercury film for the detection of Zn^2+^, Cd^2+^, Pb^2+^ and Cu^2+^ was investigated.

## 2. Experimental Section

### 2.1. Reagents

Nafion (5% wt in low aliphatic alcohols), was purchased from Aldrich, and then diluted to 1% Nafion with isopropyl alcohol. All chemicals used in this study were analytical reagent grade and used without further purification. Zn^2+^, Cd^2+^, Pb^2+^, Cu^2+^ and Hg^2+^ standard stock solutions (1,000 mg L^−1^, atomic absorption standard solution) were obtained from Sigma Aldrich and diluted as required. Sodium acetate and acetic acid were purchased from Aldrich. 0.1 M acetate buffer (pH 4.6) was used as supporting electrolyte. Ultra pure water (Millipore) was used for all preparations.

### 2.2. Apparatus

Square-wave anodic stripping voltammetry (SWASV) measurements were performed using a 797 VA COMPUTRACE instrument interfaced with a personal computer. The Nafion-G nanocomposite drop coated onto the glassy carbon electrode, served as the working electrode, with the Ag/AgCl (saturated KCl) and platinum electrode acting as the reference and auxiliary electrode respectively. All electrochemical experiments were carried out in a one compartment cell. The surface chemistries of graphite, graphite oxide and graphene were characterized on a Fourier Transform InfraRed spectrometer (Perkin Elmer Spectrum 100) and the structural properties were evaluated through X-ray diffraction (Phillips X-ray diffractometer) with Cu-Kα radiation. A tapping-mode atomic force microscope (Veeco Nanoman V) was employed to evaluate the morphology of graphite oxide and graphene, with special emphasis on estimating its thickness. The silicon tip [antimony (n) doped] had a curvature radius of 2.5–3.5 μm, a force constant of 1–5 N m^−1^ and a resonance frequency of 60–100 kHz. The samples for AFM were prepared by drop coating the graphene/water and graphene oxide/water (5 μL) dispersion onto a silicon wafer. Transmission electron microscopy images were taken on a Tecnai F20 HRTEM and the Raman spectra were recorded on a Dilor XY Raman spectrometer with a Coherent Innova 300 Argon laser with a 514.5 nm laser excitation.

### 2.3. Preparation of Graphene Solution

The graphite oxide was synthesized from graphite powder according to the literature with little modification [19,20]. Graphite oxide (100 mg) was dispersed in 100 mL of water and sonicated for 1 h, followed by the addition of 200 mg of NaBH_4_ to the dispersion. The mixture was stirred at 125 °C for 3 h. The black solid was isolated by centrifugation, washed with water and then dried.

### 2.4. Preparation of Modified Electrode

A 0.5 mg mL^−1^ graphene solution (100 μL) was mixed with an equal volume of 1.0 wt% Nafion-isopropyl alcohol solution by ultrasonication for *ca.* 30 min. or until fully dispersed. Then, a 5 μL aliquot of the mixture was coated onto the glassy carbon electrode (GC) to obtain the Nafion-G modified electrode [18].

### 2.5. Procedure for SWASV Analysis

The three electrodes were immersed into the electrochemical cell, containing 20 mL 0.1 M acetate buffer (pH 4.5), 10 mg L^−1^ Hg^2+^, and the target metal ions. The Nafion-G modified GC electrode with mercury film was plated *in situ* by spiking the sample with the required concentration of Hg^2+^ and simultaneously depositing Hg and the target metals on the surface of the electrode at −1.3 V for 120 s. Following the conditioning step, the stirring was stopped and after 10 s the voltammogram was recorded by applying a continuously changing square-wave potential (with a voltage step of 5 mV, amplitude of 25 mV, and frequency of 50 Hz). Prior to the next cycle, a cleaning step (60 s at 0.3 V, with solution stirring) was used to remove the target metals and metal film.

## 3. Results and Discussion

### 3.1. Morphology and Structural Characterization of Graphene

**Figure 1.**
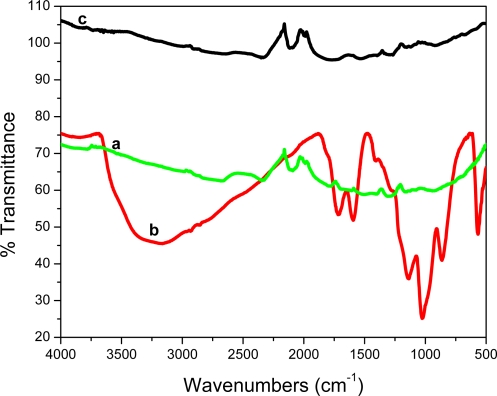
FT-IR spectra of graphite (curve **a**), graphite oxide (curve **b**) and graphene (curve **c**).

Figure 1 shows the Fourier Transform InfraRed (FT-IR) spectra of graphite, graphite oxide (GO) and graphene. For graphite, no distinct peaks are detected. GO however, showed a rich collection of transmission bands corresponding to C=O (1,719 cm^−1^), aromatic C=O (1,597 cm^−1^), carboxy C–O (1,411 cm^−1^, epoxy (1,283 cm^−1^) and O–H (3,400 cm^−1^) groups. After reduction with NaBH_4_ most of the functional groups were eliminated. These results concur with those reported by Chen *et al.* [21].

**Figure 2.**
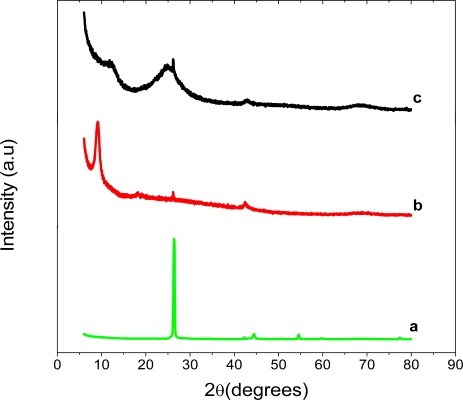
XRD patterns of graphite (curve **a**), graphite oxide (curve **b**) and graphene (curve **c**).

The XRD patterns of graphite, graphite oxide (GO) and graphene are shown in Figure 2. Graphite showed a very strong 002 peak at 26.37°, GO a 001 peak at 9.88° and graphene, 002 peak at 24.88°. The GO peak shift is due to the formation of hydroxyl, epoxy and carboxyl groups. After reduction to graphene some of the oxygen-containing functional groups are removed and this causes the graphene peak to shift to 24.88°. This suggests the conjugated graphene network (sp^2^ carbon) is reestablished during the reduction process, which is associated with the ring-opening of the epoxides.

**Figure 3.**
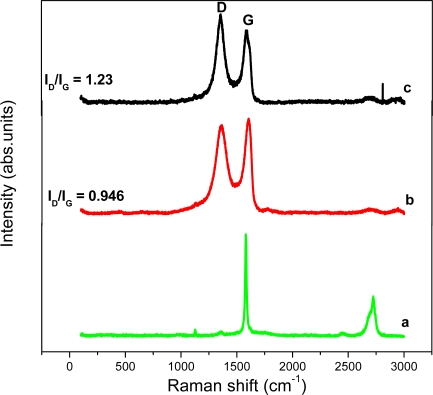
Raman spectra of graphite (curve **a**), graphite oxide (curve **b**) and graphene (curve **c**).

The Raman spectrum of graphite, GO and graphene is shown in Figure 3. The Raman spectra of the materials simply confirm the observations of the XRD patterns *i.e.*, the changes of structure during the reduction process from GO to graphene. The intensity ratio (I_D_/I_G_) of D band and G band of GO is about 0.946, while the I_D_/I_G_ of graphene is 1.23 due to the presence of unrepaired defects that remained after the removal of large amounts of oxygen-containing functional groups. This I_D_/I_G_ ratio value is consistent with most chemical reduction reports by Fan *et al.* [22].

**Figure 4.**
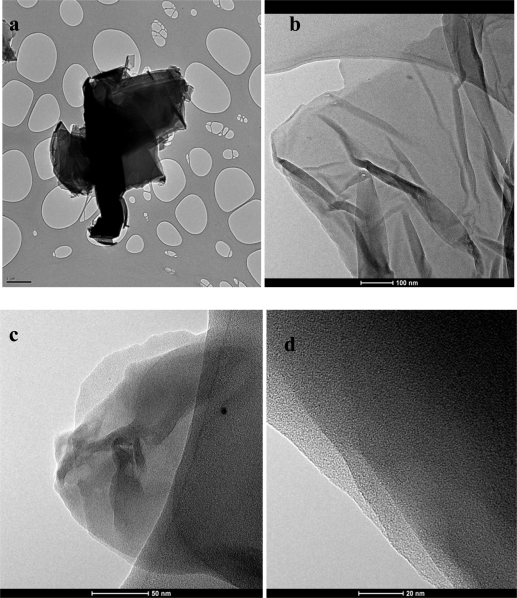
TEM images of **(a)** graphite, **(b)** graphene oxide, **(c)** graphene and **(d)** HRTEM of graphene.

For further characterization Transmission Electron Microscopy (TEM) analysis was done. TEM samples were prepared by pipetting the graphene dispersion onto a holey mesh grid. The TEM image of graphite [Figure 4(a)], shows its graphitic structure as large thick dark flakes. Graphite cannot be exfoliated even when sonicated under the same conditions as GO. For GO [Figure 4(b)] large sheets were observed to be situated on top of the grid, resembling a wavy silk veil. The sheets are transparent and entangled with one another. The structure of graphene (reduced GO) is different from that of GO [Figure 4(c)]. At low magnification, the structure of graphene looks flat, with transparent layers on top of one another. The silk-like parts as well as the restacked parts can be seen. Wrinkles and folding are also observed on the surface as well as at the edges of the structure. Corrugation and scrolling are part of the intrinsic nature of graphene nanosheets, which results from the fact that the 2-D membrane structure becomes thermodynamically stable via bending [23]. Figure 4(d) shows a high resolution TEM (HRTEM) image of graphene. Layers of graphene can be seen in this image.

**Figure 5.**
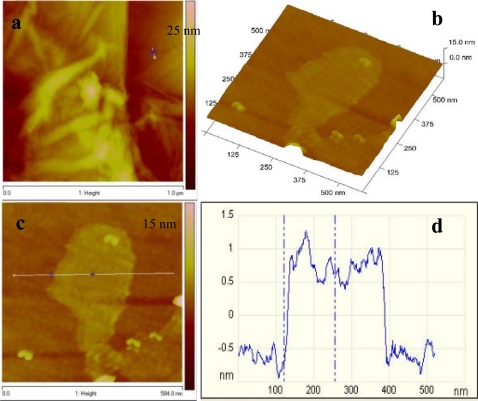
**(a)** AFM topography image of graphene, **(b)** 3D representation of the selected area in **(a)** and **(c)** line scan of the selected individual graphene.

Atomic force microscopy (AFM) was also performed on graphene, to characterize the degree of exfoliation. Figure 5(a) represents the AFM topography image of graphene, wherein several graphene sheets were randomly deposited on the silicon substrate. A flat graphene sheet was selected for further investigation using the 3D view [Figure 5(b)]. The graphene surface was slightly rough and this could be due to the existence of some functional groups. The cross sectional view across the plain area of the sheet gave an estimated height of 1.3 nm which is consistent with that reported by Shen *et al*. [24].

### 3.2. Electrochemical Characterization of the Nafion-G Nanocomposite Film

**Figure 6.**
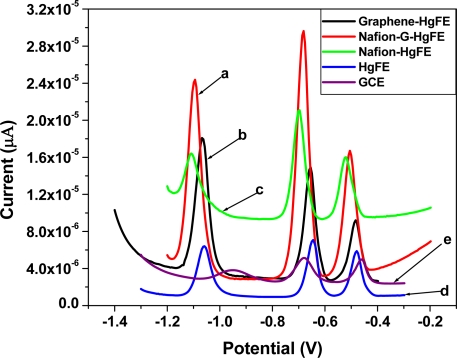
SWASV of 30 μg L^−1^ of Zn^2+^, Cd^2+^ and Pb^2+^ on **(a)** Nafion-G HgFE, **(b)** Graphene HgFE, **(c)** Nafion HgFE, **(d)** HgFE and **(e)** glassy carbon electrode in 0.1 M acetate buffer (pH 4.6).

Figure 6 shows the SWASV analytical characteristics of different film coated GC electrodes e.g., Nafion (green line), Nafion-G (solid line), Graphene (black line) by *in situ* plated Hg-film, for Zn^2+^, Cd^2+^ and Pb^2+^ determination. The stripping voltammograms were obtained in a solution containing 30 μgL^−1^ of each of the target metals, 10 mgL^−1^ Hg^2+^ in 0.1 M acetate buffer (pH 4.6). The sharper higher peak current for the target metal ions on the five metal films were obtained at the Nafion-G-modified electrode, and is consistent with the voltammetric behaviors of carbon nanotubes/Nafion [12] and ordered mesoporous carbon/Nafion electrodes [14]. The signal enhancement may be attributed to the change of the morphologies and the structure as well as the interfacial electron-transfer properties. Each peak appearing at a certain peak potential in Figure 6, represents the point at which a particular metal strips out of the amalgam (stripping step) or is re-oxidized back into solution. The stripping potentials for Zn^2+^, Cd^2+^ and Pb^2+^ appear at approximately −1.1 V, −0.7 V and −0.5 V respectively; the redox reaction involved during stripping analysis is given by Equation (1):

(1) $$\begin{array}{l} {\text{Mn}}^{n+}+né+\;\text{Hg}\;\to M\;(\text{Hg})\;\;\;\;\text{Deposition step}\;(\text{reduction reaction})\\ M\;(\text{Hg})\to M^{n+}+né+\text{Hg}\;\;\;\;\;\;\text{Stripping Step}\;(\text{oxidation reaction}) \end{array}$$

### 3.3. The Effect of Experimental Variables

**Figure 7.**
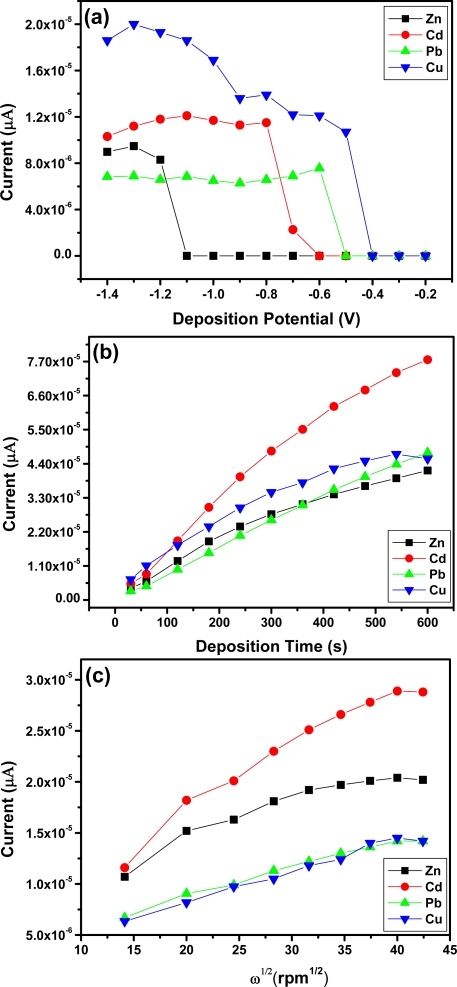
Effect of **(a)** deposition potential, **(b)** deposition time and **(c)** rotation speed on the stripping peak current of Zn^2+^, Cd^2+^, Pb^2+^ and Cu^2+^ on a Nafion-G mercury film electrode in 0.1 M acetate buffer (pH 4.5).

Figure 7(a) shows the effect of deposition potential on the peak current of Zn^2+^, Cd^2+^, Pb^2+^ and Cu^2+^ after 120 s, deposition was studied in the potential range from −0.2 V to −1.5 V. As the deposition potential shifts from −0.6 V to −1.2 V the stripping peak current increased and the more negative the peak potential became the peak currents started to reduce. The different trends observed for Zn^2+^, Cd^2+^, and Pb^2+^ and Cu^2+^ may be due to the different standard potentials. Thus −1.3 V was used as the optimal deposition potential for Zn^2+^, Cd^2+^ and Pb^2+^ in subsequent experiments whereas, an optimal deposition potential of −1.0 V was chosen for Cu^2+^.

The effect of deposition time on the peak currents of Zn^2+^, Cd^2+^, Pb^2+^ and Cu^2+^ was studied and the results obtained are shown in Figure 7(b). As the deposition time increased so did the stripping peak current of each metal ion. The increase occurred linearly with deposition time because of the increased amount of analyte on the Nafion-G modified electrode. A deposition time of 120 s was chosen for further analysis due to the rapid surface saturation which occurred after 120 s.

The effect of rotation speed during the pre-concentration step was also studied in the range 200–2,000 rpm [Figure 7(c)]. As the square-root of rotation speed of the stirring rod increased so did the stripping peak currents of Zn^2+^, Cd^2+^, Pb^2+^ and Cu^2+^ [28]. Establishing the optimum rotation speed facilitates the convective transport of the metal ions in solution to the working electrode surface and hence contributes towards the sensitivity of stripping analysis. A rotation speed of 1,000 rpm was chosen for further analysis.

### 3.4. Analytical Performance

Individual analysis of Zn^2+^, Cd^2+^, Pb^2^ and Cu^2+^

**Figure 8.**
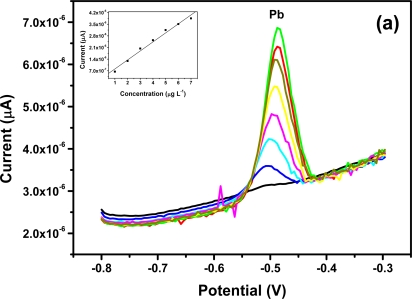

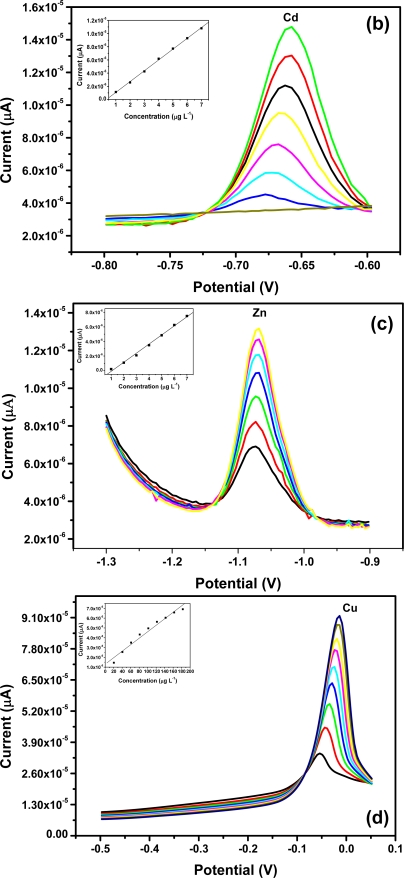

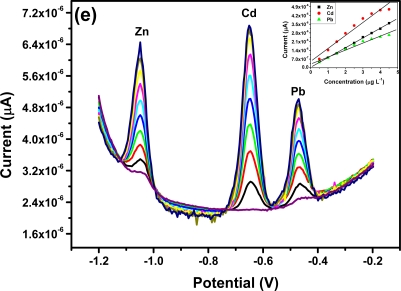
SWASVs of (**a**) Pb^2+^, (**b**) Cd^2+^, (**c**) Zn^2+^, (**d**) Cu^2+^ and simultaneous analysis of Pb^2+^, Cd^2+^ and Zn^2+^ in (**e**) in 0.1 M acetate buffer (pH 4.6).

All four metals *i.e.*, Zn^2+^, Cd^2+^, Pb^2+^ and Cu^2+^ were determined individually at the Nafion-G mercury film electrode using SWASV. Calibration plots [Figures 8(a–d)], for individual metal solutions ranging from 1–7 μg L^−1^ for Zn^2+^, Cd^2+^, Pb^2+^ and 20 μg L^−1^–180 μg L^−1^ for Cu^2+^, gave the sensitivities and detection limits shown in Table 1. A slight shift in the peak potentials of the metals with increasing metal ion concentration towards positive potential was observed and suggests an IR-drop effect. Figure 8(e) is the voltammograms for 0.5–5.0 μg L^−1^ of Zn^2+^, Cd^2+^ and Pb^2+^ at the Nafion-G-Hg film. The calibration plots [Figure 8(e)] for the simultaneous analysis of metals gave the sensitivities and detection limits shown in Table 2. Copper was not determined simultaneously with Zn^2+^, Cd^2+^ and Pb^2+^ due to the intermetallic interference which exists between Cu-Zn [25].

### 3.5. Comparison between Individual and Simultaneous Analysis

When comparing individual with simultaneous analysis, a difference in the sensitivities for some of the metals was observed. The sensitivity of Pb^2+^ remained relatively the same, whereas Cd^2+^ and Zn^2+^ showed a significant decrease when analyzed simultaneously. A similar trend was observed when Zn^2+^ was determined individually; here a sensitivity of 1.25 μA L μg^−1^ was obtained in comparison to 0.758 μA L μg^−1^ for the simultaneous determination.

In general, higher sensitivities were obtained for individual analysis, since only one of the metals binds to the limited number of active sites at the modified electrode surface and is involved in forming the amalgam film during the deposition step. However, during simultaneous analysis all metals present in solution compete for the limited number of active sites and are all co-deposited during the formation of the amalgam film. In addition, differences in sensitivities between individual and simultaneous determinations can also be attributed to the formation of intermetallic compounds between heavy metals when present together in the same solution [25]. The sensitivity of Pb^2+^ remained the same during both individual and simultaneous analysis since, it is most likely to be available for deposition whereas, the Cd^2+^ and Zn^2+^ are involved in a Zn-Cd intermetallic compound formation [26].

### 3.6. Detection Limits

The detection limits (3σ blank/slope) of the metals for both individual and simultaneous analysis were determined using a deposition time of 120 s and are summarized in Tables 1 and 2. A summary of previously reported analyses for Zn^2+^, Cd^2+^ and Pb^2+^ are shown in Table 3. The detection limits are lower for reference [18] and [39] since longer deposition times are being used to pre-concentrate the metal ions. In this work a shorter deposition time is being used, and therefore offers higher detection limits in comparison to those previously studied.

### 3.7. Application

The accuracy of the analysis at the Nafion-G modified electrode was evaluated through recovery studies. The electrolyte (0.1 M acetate buffer) was spiked with a known amount of metal ions and analyzed by SWASV using a deposition time of 120 s. Four replicates were run for each sample and their concentrations determined using the Standard Addition method. The recovery percentages of the metals are shown Table 4.

Individual analysis of Cd^2+^, Pb^2+^ and Cu^2+^ was done in the same solution whereas, Zn^2+^ was determined using a fresh sample. The reason being, that a very low recovery for Zn^2+^ was obtained when analyzed in the same sample as well as simultaneously. This is due to the competition of metals for the Nafion-G mercury film and also the intermetallic interaction which exists between Zn^2+^ and Cu^2+^. According to Lazar *et al.* [25], this interference is most pronounced when Zn^2+^ and Cu^2+^ are determined simultaneously, which is clearly shown from the results obtained (Table 4).

Results from Table 4 also show a variation in stripping voltammetry and ICP-MS analysis. This may be due to different interferences which are inherent to a particular technique and which ultimately influences the final result. Furthermore, the low ICP-MS results could also be due to the manner in which the samples were prepared.

For the purpose of practical applicability, a real water sample was collected from Edith Stephens Wetlands Park and Nafion-G HgFE was employed for the determination of Zn^2+^, Cd^2+^ and Pb^2+^ metal ions. The lake water was adjusted to pH 4.6 using sodium acetate buffer. A deposition time of 600 s was used for the analysis. The determination of metal ions in the lake water is hampered by the presence of organic compounds (e.g., humic acids) which may form stable complexes with the metal ions thus making the metal ions unavailable for analysis. In addition, these organic acids also absorb onto the surface of the working electrode thus decreasing the surface area of the electrode causing a reduction in the analytical signal. As a consequence a longer deposition time was used to obtain a signal [40]. The results obtained were compared with ICP-MS. The Nafion-G HgFE was sensitive enough to be able to detect Zn^2+^, Cd^2+^ and Pb^2+^ as shown in Table 5. However, for Cd^2+^, a higher result was obtained with ICP-MS compared to SWASV which may be due to the intrinsic difficulties of working associated with working at ultra-trace levels. The non-detection of Cu^2+^ can be attributed to the formation of stable copper complexes with organic compounds or humic acids.

## 4. Conclusions

A highly enhanced sensing platform based on the Nafion-graphene nanocomposite film was established for the individual as well as simultaneous determination of Zn^2+^, Cd^2+^, Pb^2+^ and Cu^2+^ by square-wave anodic stripping voltammetry. The nanocomposite film combining the advantages of graphene and the cation exchange capacity of Nafion enhanced the sensitivity of the target metal ions. Herein the cation exchange capacity of the Nafion and the enhanced electron conduction of graphene are combined to yield a sensing platform with enhanced sensitivity towards selected metal ions. The Nafion not only acts as an effective solubilizing agent for graphene nanocomposite but also as an antifouling coating to reduce the influence of surface-active macromolecules. The electrochemical sensing interface exhibited excellent stripping performances for trace analysis of Zn^2+^, Cd^2+^ and Pb^2+^ combining the advantages of graphene nanosheets together with the unique features of the *in situ* plating mercury film. The analytical application of the Nafion-G modified electrode was assessed by doing recovery studies followed by real sample analysis and the result for the Nafion-G-HgFE electrode was compared with the results obtained by ICP-MS. The detection limits obtained for each metal clearly showed that this technique is capable of detecting metals below the detection requirement of the Environmental Protective Agency (EPA) namely, Pb^2+^ (15 μg L^−1^), Cd^2+^ (5 μg L^−1^) and Zn^2+^ (5 mg L^−1^).

## Figures and Tables

**Table 1.** Sensitivity values, correlation coefficients (R^2^) and detection limits for Zn^2+^, Cd^2+^, Pb^2+^ and Cu^2+^ determined individually on a Nafion-G mercury film electrode.

**Electrode**	**Individual**	**Sensitivity (μA L μg^−1^)**	**R^2^**	**Detection limit (μg L^−1^)**
	Pb^2+^	0.541 ± 0.06	0.992	0.07
Nafion-G HgFE	Cd^2+^	1.64 ± 0.13	0.999	0.08
	Zn^2+^	1.25 ± 0.22	0.997	0.07
	Cu^2+^	12.95 ± 1.13	0.985	0.13

**Table 2.** Sensitivity values, correlation coefficients (R^2^) and detection limits of Pb^2+^, Cd^2+^ and Zn^2+^ determined simultaneously on a Nafion-G mercury film electrode.

**Electrode**	**Simultaneous**	**Sensitivity (μA L μg^−1^)**	**R^2^**	**Detection Limit (μg L^−1^)**
	Pb^2+^	0.557 ± 0.04	0.990	0.07
Nafion-G HgFE	Cd^2+^	1.070 ± 0.10	0.983	0.13
	Zn^2+^	0.758 ± 0.07	0.999	0.14

**Table 3.** Summary of work done previously on Zn^2+^, Cd^2+^ and Pb^2+^ on various electrodes.

**Detected Metal**	**Electrode Type**	**Deposition Time**	**Electrochemical Stripping Technique**	**Detection Limit (μg L^−1^)**	**Reference Number**
Pb^2+^, Cd^2+^	Sb film C-paste	120 s	SWASV	Pb^2+^ = 0.8 Cd^2+^ = 0.2	[27]
Pb^2+^, Cd^2+^, Zn^2+^	Bi-C-nanotube	300 s	SWASV	Pb^2+^ = 1.3 Cd^2+^ = 0.7 Zn^2+^ = 12	[28]
Pb^2+^, Cd^2+^	Bi film C-paste	120 s	SWASV	Pb^2+^ = 0.8 Cd^2+^ = 1.0	[29]
Pb^2+^, Cd^2+^	Bi nanopowder on carbon	180 s	SWASV	Pb^2+^ = 0.15 Cd^2+^ = 0.07	[30]
Pb^2+^, Cd^2+^, Zn^2+^	Bi/poly (p-ABSA)	240 s	DPASV	Pb^2+^ = 0.8 Cd^2+^ = 0.63 Zn^2+^ = 0.62	[31]
Pb^2+^, Cd^2+^, Zn^2+^	Bi nanoparticles on screen printed C	120 s	SWASV	Pb^2+^ = 0.9 Cd^2+^ = 1.3 Zn^2+^ = 2.6	[32]
Pb^2+^, Cd^2+^, Zn^2+^, Cu^2+^	Boron-doped diamond	60 s	DPASV	Pb^2+^ = 1.15 Cd^2+^ = 0.36 Zn^2+^ = 1.6	[33]
Pb^2+^, Cd^2+^, Zn^2+^	Disc graphite BiFE	120 s	SWASV	Pb^2+^ = 0.497 Cd^2+^ = 0.325 Zn^2+^ = 0.785	[34]
Pb^2+^, Cd^2+^, Zn^2+^, Cu^2+^, Ag^+^	Boron-doped diamond		DPASV	Pb^2+^ = 5.0 Cd^2+^ = 1.0 Zn^2+^ = 50	[35]
Pb^2+^, Cd^2+^, Zn^2+^, Cu^2+^, Ag^+^	Mercury film electrode		DPASV	Pb^2+^ = 5.0 Cd^2+^ = 1.0 Zn^2+^ = 10	[35]
Pb^2+^, Cd^2+^	BiFE	90 s	SWASV	Pb^2+^ = 6.9 Cd^2+^ = 1.4	[36]
Pb^2+^, Cd^2+^, Zn^2+^	NC(Bpy)BiFE	120 s	SWASV	Pb^2+^ = 0.077 Cd^2+^ = 0.12 Zn^2+^ = 0.56	[37]
Pb^2+^, Cd^2+^, Zn^2+^	NC BiFE		SWASV	Pb^2+^ = 2 Cd^2+^ = 2 Zn^2+^ = 6	[38]
Cd^2+^	Nafion-G HgFE	500 s	DPASV	Cd^2+^ = 0.005	[18]
Pb^2+^, Cd^2+^	Nafion-G BiFE	300 s	DPASV	Pb^2+^ = 0.02 Cd^2+^ = 0.02	[39]
Pb^2+^, Cd^2+^, Zn^2+^	Nafion-G HgFE	120 s	SWASV	Pb^2+^ = 0.04 Cd^2+^ = 0.08 Zn^2+^ = 0.07	In this work

**Table 4.** Recovery studies of metals determined on the Nafion-G mercury film electrode compared with ICP-MS.

**Electrode**	**Individual**	**Simultaneous**	**ICP-MS**
Pb^2+^	100.2%	78.5%	72.0%
Cd^2+^	113.9%	90.8%	71.2%
Zn^2+^	69.0%	19.8%	94.2%
Cu^2+^	89.0%	64.0%	84.1%

**Table 5.** Analysis of Lake Water using Nafion-G HgFE *vs.* ICP-MS.

Metals	Individual (μg L^−1^)	ICP-MS (μg L^−1^)
Pb^2+^	0.534 ± 0.042	0.520 ± 0.01
Cd^2+^	0.1403 ± 0.005	<0.009 ± 2.9 × 10^−5^ – 0.65 ± 0.02
Zn^2+^	1.817 ± 0.499	2.301 ± 0.03
Cu^2+^	Not Detected	5.780 ± 0.08
